# Exchanging Genes Within a City: Analysis of Pollen Flow Patterns in a Narrow Endemic Plant Species Threatened by Urbanisation

**DOI:** 10.1002/ece3.73406

**Published:** 2026-04-13

**Authors:** Nicola Delnevo, Andrea Piotti, Eddie J. van Etten, William D. Stock, David L. Field, Margaret Byrne

**Affiliations:** ^1^ Edith Cowan University Joondalup Western Australia Australia; ^2^ Biodiversity and Conservation Science, Department of Biodiversity, Conservation, and Attractions Bentley Delivery Centre Bentley Western Australia Australia; ^3^ Institute of Biosciences and BioResources (IBBR), National Research Council (CNR) Sesto Fiorentino Italy; ^4^ Applied Biosciences Macquarie University Sydney New South Wales Australia; ^5^ University of Western Australia Perth Western Australia Australia

**Keywords:** biodiversity hotspot, *Conospermum*, habitat fragmentation, native bees, paternity analysis, Proteaceae

## Abstract

Human‐driven habitat fragmentation is known to disrupt gene flow in a wide range of species. However, its consequences for endemic plant species that depend on specialised invertebrate pollinators remain poorly understood. Although honeybees are capable of travelling considerable distances while foraging, many small‐bodied native bee species lack the capacity to expand their foraging ranges or cross anthropogenic barriers, rendering habitat fragmentation the most significant threat to these ecological interactions. In many long‐lived plants, the time since fragmentation has not been long enough for detectable changes in the genetic composition of standing populations. Changes in dispersal patterns observed in seedlings may thus provide insights into future impact of habitat changes. Using paternity assignment analysis, we characterised the plant mating system and changes in dispersal patterns across multiple fragmented and non‐fragmented populations. Results showed that while pollen could travel unimpeded through unfragmented bushland, fragments separated by built‐up areas resulted in complete isolation, with no pollen immigration. Extensive landscape fragmentation for urbanisation limited the capacity of native pollinators to maintain effective inter‐population pollen flow. Reduced contemporary gene flow may limit the capacity of small populations to regain lost genetic diversity via connectivity with larger habitat remnants, potentially compromising the long‐term persistence of threatened species that depend on specialised pollinators. These findings underscore the need to incorporate species‐specific life history characteristics and ecological requirements into conservation planning, including translocation and reintroduction efforts, to ensure populations are large enough to maintain genetic diversity and functional mating systems within populations.

## Graphical abstract

Anthropogenic habitat fragmentation can severely disrupt gene flow in endemic plants that depend on species‐specific native pollinators with limited dispersal ability. Using paternity analysis across fragmented and continuous populations, we found that pollen flow was maintained in intact bushland but was completely absent between fragments separated by urban infrastructure, indicating strong contemporary isolation. These findings suggest reduced capacity for genetic rescue in small populations and underscore the need to incorporate species‐specific pollination ecology into conservation planning.

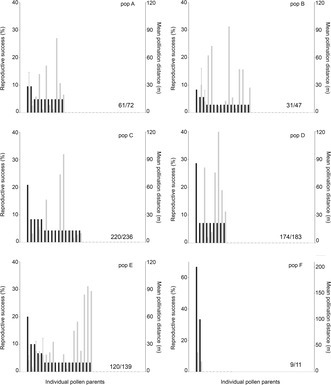

## 1 Introduction

Global human populations are increasingly concentrated in densely populated urban environments, leading to the rapid expansion of cities as a dominant habitat type worldwide (Grimm et al. 2008). Despite urban and peri‐urban areas occupying only around 3% of the Earth's terrestrial surface (Faeth et al. 2011), the associated anthropogenic fragmentation of habitats represents a major contributor to the ongoing biodiversity crisis, with consequences for both human societies and the organisms reliant on intact natural ecosystems (Haddad et al. 2015; Des Roches et al. 2021). Organisms living in urban settings are exposed to a suite of unique stressors that can hinder the long‐term viability of their populations (Beninde et al. 2018; González et al. 2019). For many organisms in urban environments, increased dissection of original habitat has reduced dispersal between fragments as well as population sizes. Given the increased pace of habitat modification, further research is required across diverse organisms with different life histories and dispersal capabilities to better understand the factors that negatively impact population viability in urban environments.

Given the sessile nature of plants, land clearing and man‐made infrastructure are often thought to generate barriers to gene flow among population fragments. Over time this may lead to genetic isolation and, consequently, greater fragmentation and reduction in size of originally more contiguous populations (Johnson and Munshi‐South 2017; Miles et al. 2019; Schmidt et al. 2020). Barriers to gene flow between population fragments are expected to affect the patterns of mating and pollen dispersal, with differing impact depending on the response of specific pollinators to rapid habitat change (Aguilar et al. 2019; Brudvig et al. 2015; Schmidt et al. 2020; Wenzel et al. 2020). Birds and European honeybees, which are capable of long‐distance movement, frequently adjust to wider plant spacing by expanding their foraging range (Beekman and Ratnieks 2000; Saunders and De Rebeira 1991). Many plant species that rely on these vectors have shown extensive, or even increased gene flow among populations following habitat loss and fragmentation (Bezemer et al. 2016; Byrne et al. 2007, 2008). Honeybees ( *Apis mellifera* ) have been introduced to many areas worldwide and are considered to be particularly effective pollinators in modified landscapes owing to their high population densities and eusocial structure (Dick et al. 2003). In contrast, numerous native plant species depend on highly specialised pollination relationships. This is particularly evident in Australia, where the native bee assemblage is large and diverse, and differs from those found on other continents (Houston 2018). Consequently, findings from studies examining honeybee responses to habitat fragmentation and their effects on pollination dynamics cannot be readily applied to plant lineages of ancient Gondwanan origin, such as the Proteaceae. The prolonged evolutionary isolation of Australia's flora, spanning at least 34 million years (Exon et al. 2006; McLoughlin 2001), has promoted the evolution of specialised pollination systems and distinctive floral morphologies. Although numerous iconic proteaceous taxa, such as *Banksia* and *Grevillea*, have coevolved with birds as their primary pollinators, insect‐mediated pollination also plays a critical role in Australian ecosystems. Many taxa within this ancient plant family depend entirely on native insects for successful pollination (Hopper and Gioia 2004). The high specificity of these plant–insect mutualisms highlights the importance of assessing how habitat fragmentation affects such interactions, especially for effective conservation management.

Population genetic theory predicts that small and isolated plant populations can be prone to genetic decline through demographic bottlenecks and genetic drift (Ellstrand and Elam 1993). It is widely accepted that declines in genetic diversity in such populations can have negative effects on seed production and progeny fitness (Aguilar et al. 2019; González et al. 2019). Nonetheless, a number of syntheses of the empirical literature have revealed mixed responses to decline in populations size and isolation (Aguilar et al. 2019; Lowe et al. 2015; Barrett and Harder 2017). One possible explanation lies in the complex interactions with life history of individual species that mediate the pace of population genetic change following habitat fragmentation. For example, populations of long‐lived plants have often experienced recent fragmentation events relative to lifespan, making it difficult to detect the genetic effects, in particular when the investigation is focused on adult trees (Lowe et al. 2015). In this regard, the amount of current (post‐fragmentation) pollen dispersal, and the distance over which it occurs, have been proposed as possible early signals. Such early signals are likely to be more readily detected in the short‐term than changes in genetic diversity metrics, yet they are also expected to play a significant role in shaping the future genetic structure and reproductive processes in fragmented populations (e.g., Dubois and Cheptou 2017; Lowe et al. 2015; Robledo‐Arnuncio et al. 2014). For many flowering plants, the majority of which rely on pollination by biotic vectors (Ollerton et al. 2011), the long‐term functioning of fragmented populations is dependent on the responses of pollinators to the changes in the spatial arrangement of remnant plants.

*Conospermum* (Proteaceae) is an insect‐pollinated genus endemic to Australia with its greatest diversity occurring in the south‐west Australian biodiversity hotspot. In this region, *Conospermum* species have long been used by the Noongar people for medicinal purposes (Decosterd et al. 1993; Janke 1998, 2018), and several taxa are also commercially harvested for the cut‐flower industry. The genus comprises 53 recognised species (Bennett 1995), four of which are currently listed as threatened under Western Australian legislation (W.A. Government Gazette 2018). Among these, *Conospermum undulatum* is a long‐lived lignotuberous shrub currently threatened by urban development, with its entire distribution being now completely embedded within a major urban area. This species has coevolved to facilitate pollination by the small native bee *Leioproctus conospermi* , together with native ants as secondary pollinators (Delnevo, van Etten, Clemente, et al. 2020; Delnevo and van Etten 2019). Like most species within the genus, *C. undulatum* possesses characteristic flowers that appear too small to be effectively pollinated by introduced European honeybees, and therefore represents an opportunity to study patterns of pollen flow in species that rely exclusively on native pollinators. Recent research has demonstrated that habitat fragmentation adversely affects the reproductive success of *C. undulatum* , reducing both seed production and germination rates (Delnevo, van Etten, Byrne, et al. 2020). Interestingly, genetic characteristics alone showed a weak effect on the reproduction of the species, and this was attributed to the fact that population fragmentation was too recent to show the predicted effect of genetic drift on the adult cohorts of plants analysed (Delnevo et al. 2021).

Characterising plant mating systems and changes in dispersal patterns in fragmented and non‐fragmentated landscapes is an important part of understanding the impacts of urbanisation on plant populations. In many long‐lived plants, such as *Cononsperumum*, where the time since fragmentation has not been long enough for detectable changes in the genetic composition of standing populations, changes in dispersal patterns observed in seeds may provide insights into future impact of habitat changes. In this study, we aimed to investigate these early signals of the effects of fragmentation on the threatened species *C. undulatum* that relies on small native pollinators for reproduction. Specifically, we used paternity assignment analysis to: (i) quantify the amount of pollen immigration into different populations, (ii) determine the distances over which local pollen dispersal occurred and (iii) identify the conservation implications of the observed post‐fragmentation patterns of pollen dispersal.

## 2 Materials and Methods

### 2.1 Study Species and Area

*Conospermum undulatum* (Proteaceae) is a long‐lived species that can survive at least several decades as individual shrubs are able to resprout from semi‐subterranean woody lignotubers after disturbance such as fire or mechanical damage from strong winds (Bennett 1995). During the flowering season (late August‐ late October) it produces white inflorescences. *Conospermum undulatum* is an entomophilous species and has a specific suite of native insect pollinators, with the small specialised native bee *Leioproctus conospermi* being the most active floral visitor (Delnevo, van Etten, Byrne, et al. 2020). Indirect estimates of genetic connectivity suggests gene flow is mantained for up to 1000 m by this native pollinators through non‐fragmented landscapes (Delnevo et al. 2021). Flowers of *C. undulatum* have a characteristic pollination mechanism that involves a tactile stimulation within the calyx. When an insect visits, the pressure applied by its mouthparts at the base of the style causes it to flick away from the fertile anthers and make contact with the visitor. This action presses the moist, cup‐shaped stigma onto the pollinator, collecting any pollen it carries, while simultaneously triggering the explosive release of pollen from the fertile anthers onto the insect. (Douglas 1997). This remarkable pollination system is an effective physical barrier that prevents autogamous selfing. Recent flower manipulation experiments indicated that self‐incompatibility in *C. undulatum* arises not only from its specialised floral morphology, which prevents autogamy, but also from a genetic mechanism consistent with early‐acting inbreeding avoidance, preventing self‐pollination between flowers on the same plant (Delnevo, van Etten, et al. 2019). *Conospermum undulatum* fruits are cone‐shaped achenes (i.e., containing only one seed) that fall to the ground when mature and appear to have a spatially limited dispersal, mainly driven by gravity (Close et al. 2006; Delnevo et al. 2021). Low fecundity is common in resprouter species in the Proteaceae (Lamont et al. 2011), and seed set in *C. undulatum* is low, especially when compared with the number of flowers produced.

*Conospermum undulatum* is found in the Swan Coastal Plain (SCP) bioregion, a low‐lying coastal area stretching from Jurien Bay, north of Perth, to Cape Naturaliste in the south. This area lies within of the south‐west Australia global biodiversity hotspot (Mittermeier et al. 2004). The SCP has undergone extensive land clearing, primarily driven by urban expansion around the capital city of Perth. Currently, only about 35% of the native vegetation on the SCP remains, with just 10% protected within conservation reserves (Wardell‐Johnson et al. 2016). *Conospermum undulatum* has been impacted by land clearing for urban expansion and is currently restricted to 17 populations ranging in size from a few individuals to several hundred plants across a restricted distribution of 55 km^2^. *Conospermum undulatum* is listed as ‘vulnerable’ among the threatened flora of Western Australia using IUCN red list criteria (Department of Environment and Conservation 2009; W.A. Government Gazette 2018).

All known populations of *C. undulatum* were intensively surveyed at the beginning of the flowering season 2017. The present study focused on eight populations (Table 1). Three of these were medium‐sized populations, ranging from 139 to 236 adult plants as determined by direct counts; three were small populations, with 8, 10 and 11 plants; and two were large populations with an estimated size of several hundred plants, one of which (population A) is part of an intact nature reserve. In the larger populations, we limited our sampling to focal areas approximately 70–100 m in radius, matching the size of the smaller population fragments to allow comparison of patterns of pollen movement of smaller populations with those of large intact populations. All populations were discrete and clearly separated by cleared land. All selected populations, except populations C and H, were within the 1000 m distance that can potentially be covered by the main pollinator, *L. conospermi* (Table 2). To accurately assess pollen dispersal distances and spatial genetic structure, a BE‐3300 GNSS Surveyor (Bad Elf, West Hartford, CT, USA) was used to obtain GPS coordinates of each plant to an accuracy of 1 m.

**TABLE 1:** Genetic diversity parameters of *Conospermum undulatum* populations.

Pop	Census size	*N*	Na	*PA*	Ar_16_	PAr_16_	*H*_O_	*H*_E_	*F*_IS_	*G*_ST_	Jost's *D*
A	800	72	11.29	7	6.11	0.50	0.645	0.736	0.115	0.030	0.133
B	520	47	10.50	5	6.17	0.57	0.658	0.715	0.075	0.031	0.133
C	236	236	10.64	3	5.69	0.26	0.604	0.723	0.167	0.041	0.185
D	183	183	14.29	25	6.30	0.68	0.674	0.739	0.096	0.028	0.124
E	139	139	13.00	14	6.01	0.41	0.631	0.726	0.134	0.027	0.117
F	11	11	7.29	1	6.34	0.48	0.649	0.680	0.020	0.029	0.122
G	10	10	4.00	0	3.81	0.12	0.550	0.569	0.081	0.058	0.228
H	8	8	4.00	1	4.00	0.22	0.563	0.471	−0.163	0.089	0.307

Abbreviations: Ar_16_, allelic richness; *F*_IS_, fixation index; *G*_ST_, mean G_ST_ value between each population and all others; *H*_E_, expected heterozygosity; *H*_O_, observed heterozygosity; Jost's *D*, mean *D* value between each population and all others; *N*, sample size; Na, average number of alleles; PA, private alleles; PAr_16_, private allelic richness.

**TABLE 2:** Mating system parameters calculated from seed crops sampled from six populations of *Conospermum undulatum*.

Pop	Nearest unsampled plants (m)	Maternal plants	Progeny per mother	Mean pollen dispersal distance^b^ (m)	Model	Pollen immigration (*m*_p_)	Selfing (*s*)	AIC^c^
A^a^	—	7	3.50 (0.99)	25.12 (6.55)	*m*_p_ + *s*	0.518 (0.124)	0.034 (0.055)	2105.5
					*m*_p_ only	0.516 (0.123)	—	2103.7
					*Cervus*	0.524	0.000	—
B^a^	—	14	2.57 (1.05)	27.14 (6.64)	*m*_p_ + s	0.131 (0.083)	0.112 (0.074)	1851.2
					*m*_p_ only	0.238 (0.100)	—	1907.2
					*Cervus*	0.150	0.100	—
C	1680	11	2.18 (0.52)	35.18 (11.83)	*m*_p_ + *s*	0.102 (0.102)	0.458 (0.109)	2220.6
					*m*_p_ only	0.494 (0.126)	—	2432.3
					*Cervus*	0.000	0.417	
D	650	8	1.75 (0.49)	35.28 (12.59)	*m*_p_ + *s*	0.144 (0.095)	0.499 (0.134)	648.1
					*m*_p_ only	0.359 (0.129)	—	715.4
					*Cervus*	0.142	0.071	—
E	570	10	3.00 (0.77)	26.45 (5.10)	*m*_p_ + *s*	0.000 (0.089)	0.101 (0.055)	3325.7
					*m*_p_ only	0.125 (0.084)	—	3364.9
					*Cervus*	0.000	0.100	—
F	600	2	1.50 (0.50)	30.98 (5.77)	*m*_p_ + *s*	0.000 (NE)	0.000 (NE)	203.4
					*m*_p_ only	0.000 (0.285)	—	—
					*Cervus*	0.000	0	—
G	800	10	0		—	—	—	—
H	3900	7	0		—	—	—	—

*Note:* Pollen immigration and selfing rates were estimated by modelling two different scenarios using NMπ and by direct estimate from paternity assignment data in *Cervus*. Parameters are expressed as mean values per population. Standard errors are in parentheses.

Abbreviation: NE, not estimable.

^a^ Within focus area.

^b^ For within population outcrosses.

^c^ Akaike information criterion.

### 2.2 Population Variables

Population size in each fragment was determined by directly counting individuals, except in the two large populations, where estimates were derived from plant density and fragment area. A connectivity index was calculated using a modified incidence function model (Hanski 1994), following the methodology of Delnevo, van Etten, et al. (2019). This approach incorporates both the distances to all neighbouring populations (including those not sampled in this study) and their respective areas, providing a more accurate measure of connectivity in highly fragmented landscapes than nearest‐neighbour or buffer‐based methods (Moilanen and Nieminen 2002). Temporal isolation was assessed recently by monitoring reproductive phenology and was found to be negligible (Delnevo, van Etten, et al. 2019). The area covered by each population was obtained with ArcGIS (ESRI, Redlands, USA) using the minimum convex polygon method, and plant density was calculated.

Following disturbance, *C. undulatum* individuals regenerate through resprouting from the lignotuber to produce additional stems, and plants that go through more regeneration cycles have more stems than plants that have fewer regeneration cycles. Since fire in the area is fairly regular due to planned hazard reduction burning occurring, the number of stems can be used as a proxy for age; that is, plants with more stems are older than plants with fewer stems. Therefore, we counted the number of stems of each individual in all the investigated populations to calculate the average number of stems per population and obtain an overall estimate of population age.

### 2.3 Sampling and Genotyping

Leaf samples were collected from each plant in all the eight populations in accordance with licence restrictions to collect biological material from threatened species. Following intensive surveys, we sampled all individuals in small and medium‐sized populations to assign/exclude plants within populations as fathers. Similarly, all plants were sampled from within the selected focus areas in the large populations. We obtained a total of 706 samples that were stored in individual paper bags on silica gel before DNA extraction. Due to the expected low seed production and germination in *C. undulatum* (Delnevo, van Etten, et al. 2019), we maximised fruit collection for inclusion in the study, resulting in a total of 1206 fruits. All collected achenes were assessed for viability, and 768 viable seeds were retained. Seed germination followed the protocol as outlined in Delnevo, van Etten, et al. (2019). Although this protocol represents the best currently available method for this species, germination rates remain low, averaging 17.1% of the viable seeds. A total of 131 seedlings were obtained from 52 maternal plants representing the product of the 2017 flowering season. Two of the small populations H and G produced zero and two viable seeds, respectively, neither of which germinated. The number of seedlings per population ranged from 3 to 35 (mean = 21.7, SD = 11.9) and the number of seedlings genotyped per maternal plant ranged from 1 to 16 (mean = 2.41, SD = 2.28). These seedlings represent the full complement of germinated seeds produced by the selected maternal plants during the 2017 flowering season (i.e., no subsampling was undertaken).

Genomic DNA was extracted from adult leaf tissue, and from entire seedlings harvested 1–2 weeks after germination. Genotyping was undertaken for 19 microsatellite loci that have been developed for *C. undulatum* , as described in Delnevo, Piotti, et al. (2019). All allele bins were manually defined, and allele assignments were carefully checked and corrected as needed. Samples that failed to amplify clearly or displayed rare alleles were re‐amplified, and DNA was re‐extracted when necessary.

Deviations from Hardy–Weinberg equilibrium (HWE) and linkage disequilibrium (LD) between all pairs of loci were evaluated using GENEPOP 4.0 (Rousset 2008). Standard diversity indices, including number of alleles (Na), private alleles (PA), observed heterozygosity (HO), expected heterozygosity (HE), inbreeding coefficient (FIS), allelic richness (Ar) and rarefied allelic richness (PAr), were calculated with GenAlEx 6.5 (Peakall and Smouse 2012) and HP‐RARE (Kalinowski 2005). As a precaution, five loci (Cu15, Cu17, Cu29, Cu41 and Cu45) that potentially harboured null alleles, as identified using the Bayesian method in INEST v2.2 (Chybicki and Burczyk 2009), were excluded from these analyses. Genetic fixation and differentiation among populations were quantified using *G*_ST_ (Nei 1977) and Jost's D (Jost 2008) in GenAlEx 6.5, with significance assessed via 999 permutations.

### 2.4 Contemporary Pollen Dispersal

Although recently developed mating models are powerful tools for estimating mating system and gene flow parameters, the use of multiple methods is recommended in the literature (Bacles and Ennos 2008; Jones et al. 2010). In all the populations that produced seedlings, paternity analysis between adult plants and progeny was therefore performed on microsatellite data using two different approaches to control for the robustness of gene flow estimates. First, we used NMπ version 1.2 (Chybicki 2018) to model paternity assignment probabilities based on the spatially explicit neighbourhood model of Adams and Birkes (1991), which simultaneously produces maximum‐likelihood estimates of several mating and dispersal parameters. Second, we used *Cervus* version 3.0.7 (Kalinowski et al. 2007; Marshall et al. 1998) to perform a maximum‐likelihood categorical paternity assignment, including the correction to accommodate genotyping inconsistencies.

An advantage of NMπ over traditional parentage methods is that seed and pollen dispersal parameters, selfing rates, seed‐ and pollen‐mediated gene flow and their confidence intervals are jointly estimated. Whereas in the *Cervus* approach, paternity is investigated in subsequent steps (see Jones et al. (2010) for a comprehensive discussion on this issue). Nevertheless, when datasets have high exclusion probabilities and adequately represent potential pollen donors, paternity analysis can reveal patterns of fine‐scale pollen dispersal within populations.

We employed NMπ to estimate selfing rates (s) and pollen immigration rates (*m*_p_), along with their confidence intervals, using the default neighbourhood size and simultaneously allowing maximum‐likelihood estimation of null allele frequencies. Even if selfing was not expected based on previous observations for this species (Delnevo, van Etten, et al. 2019), we ran two different models: one with *m*_p_ only and no selfing allowed, and the other allowing for self‐pollination (*m*_p_ + *s*). Model selection was performed using the Akaike Information Criterion (AIC), and the model with the lowest AIC value was used. Nevertheless, the relatively small progeny arrays available for some populations may limit the precision of estimates of pollen immigration (*m*_p_) and selfing (*s*), potentially increasing their variance, and should therefore be interpreted with appropriate caution.

Before performing the paternity analysis in *Cervus*, 100,000 simulations were run to estimate the LOD thresholds above which paternity was assigned at the 95% (strict) and 80% (relaxed) confidence levels. The parameters for the simulations were: 0.005 as mistyping rate, the number of individuals in each population as probable candidate fathers in each respective population, 0.99 as proportion of sampled fathers, 10 as minimum number of typed loci, and self‐fertilisation allowed. After paternity was determined, selfing and pollen immigration rates were calculated directly from the paternity assignment results and compared with the corresponding estimates obtained using NMπ. Pollen immigration detected within focal areas located in large populations most likely reflects immigration from unsampled individuals within the same continuous population rather than from external populations. However, these estimates provide a baseline of pollen movement under relatively intact conditions, enabling comparison with fragmented populations (e.g., Llorens et al. 2012).

We then determined the distributions of pollination distances for the six populations that produced seedlings. Pollination distances were measured as the Euclidean distance between assigned pollen donors and maternal trees, with selfed progeny assigned a distance of 0 m. Within‐population pollen movements were then analysed by evaluating the distances over which successful pollen was transferred from paternal to maternal plants.

## 3 Results

### 3.1 Genetic Diversity

The levels of genetic diversity were mostly similar across populations, with the exception of the small populations F, G and H (Table 1). In particular, populations G and H showed the lowest values of allelic richness (Ar_16_) and heterozygosity (*H*_O_) and generally, the highest differentiation from the other populations (Table 1; pairwise population matrices of *G*_ST_ and *D* in Tables S1 and S2, respectively). Moreover, population G was the only population without private alleles.

### 3.2 Selfing Rates and Pollen Immigration

The NMπ model that included both pollen immigration (*m*_p_) and selfing (*s*) outperformed the model that only included pollen immigration (Table 2). Identification of the presence of selfing was surprising given the expectation of *C. undulatum* being self‐incompatible, suggesting that the transfer of self‐pollen between different flowers of the same plant can, in some cases, lead to the production of viable seeds. The results from the NMπ model were also supported by direct estimates from paternity assignment. The selfing rate in the largest populations A and B was low, as well as in population E, which has less than 200 plants, compared to the high rates detected in the medium‐sized populations C and D (Table 2). The selfing rate in the small population F was zero (Table 2). Interestingly, population B had a single plant that produced an unexpectedly high number of selfed progeny (13 selfed seeds out of a total of 16). This plant has gone through numerous regeneration cycles (11 stems) and was a clear outlier in relation to the selfing rate. Therefore, we ran the neighbourhood model without this plant. Overall, plants that sired selfed progeny had a higher average number of stems (7.54 ± 0.86) compared to the mean across all *C. undulatum* individuals, which was 4.77 ± 0.17 stems. Pollen immigration rates were high in the largest population (A), which has approximately 800 plants, but rates were similar to other moderate‐sized populations for the second largest population (B, ~520 plants; Table 2). Population C, despite being the third largest population with 236 plants, had a non‐significant pollen immigration rate (0.000 from direct estimate after paternity assignment with *Cervus*; Table 2). The two populations with between 100 and 200 plants showed differing responses, with similar pollen immigration to larger population B detected in population D, but no pollen immigration detected in population E. Small populations did not produce any seeds with the exception of population F that showed no pollen immigration (estimates for population F are generated from only three seedlings).

Direct estimates of pollen immigration and selfing rates from paternity assignment with *Cervus* were consistent with the ones obtained using the neighbourhood model (Table 2). The only exception was population D, for which NMπ gave a selfing rate of 0.499 (± 0.134 SE), whereas direct calculation from *Cervus* resulted in a selfing rate of 0.071. This discrepancy was due to NMπ assigning three seeds of a mother plant as selfed progeny, while *Cervus* assigned the paternity of those seeds to a nearby plant (only 0.97 m away), with selfing being the second‐most likely assignment. These two plants in close proximity were closely related, differing by one allele at a single locus, thus generating the different paternity assignment.

**FIGURE 1:**
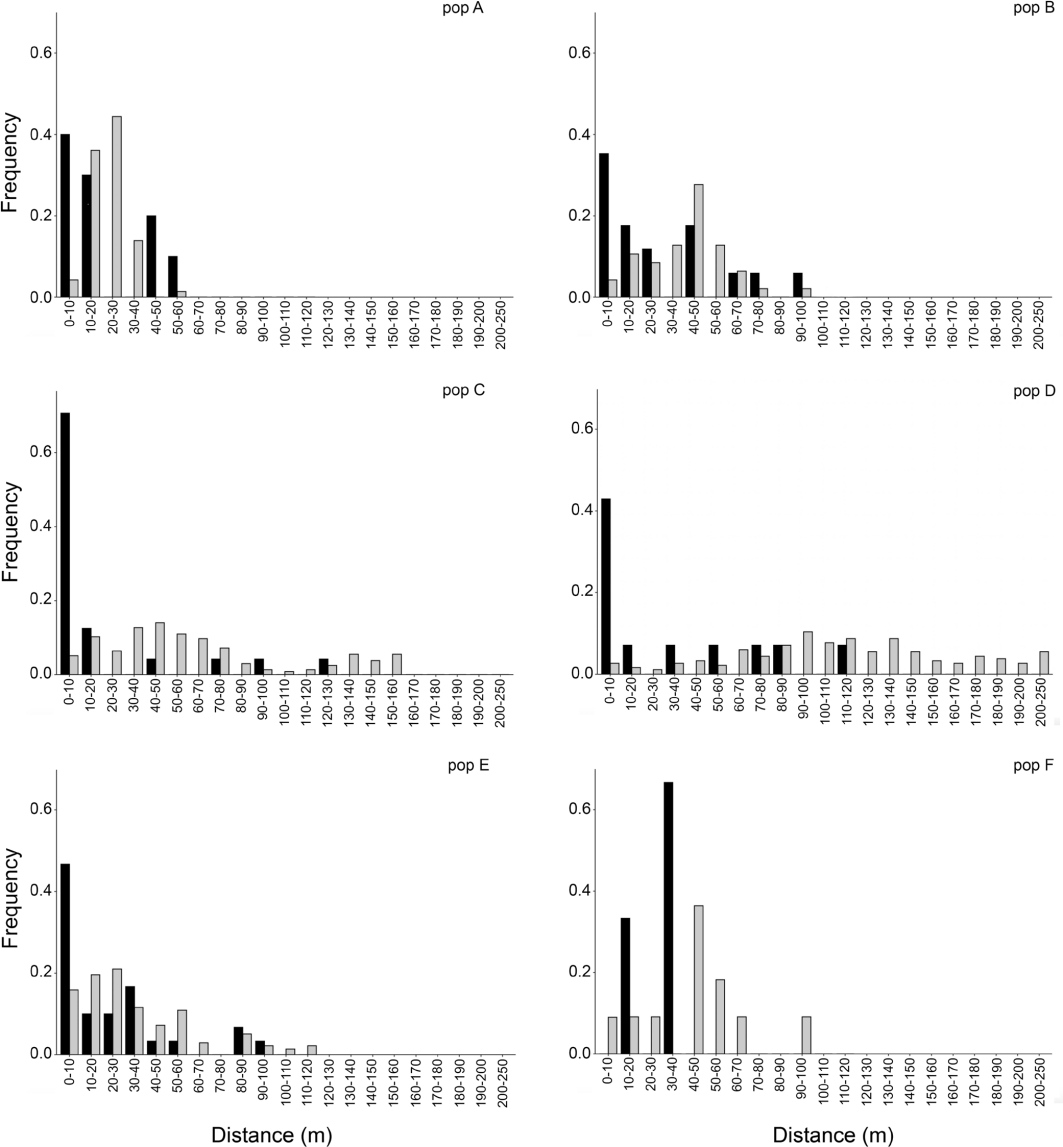
Frequency distribution of inferred pollination distances (black bars) and interplant distances of all plants relative to sampled seed mothers (grey bars).

The overall dispersal distances for outcrossed pollination events detected by the paternity assignment ranged between 0.34 m and 125.04 m, with a median distance of 16.80 m and a mean of 28.96 m (30.24 SD). The distributions of pollen dispersal distances were generally lower than the expected distributions considering the potential distances among all possible pollen donors and maternal plants (Figure 1). This trend was less evident in the two large populations A and B (Figure 1) that also had lower frequencies of local fathers within the first 10 m from the mother plants. In particular, population C had the most skewed distribution of dispersal distances compared to the other medium populations D and E, with only a few successful pollination events over 20 m from the mother plant.

### 3.3 Individual Reproductive Success

**FIGURE 2:**
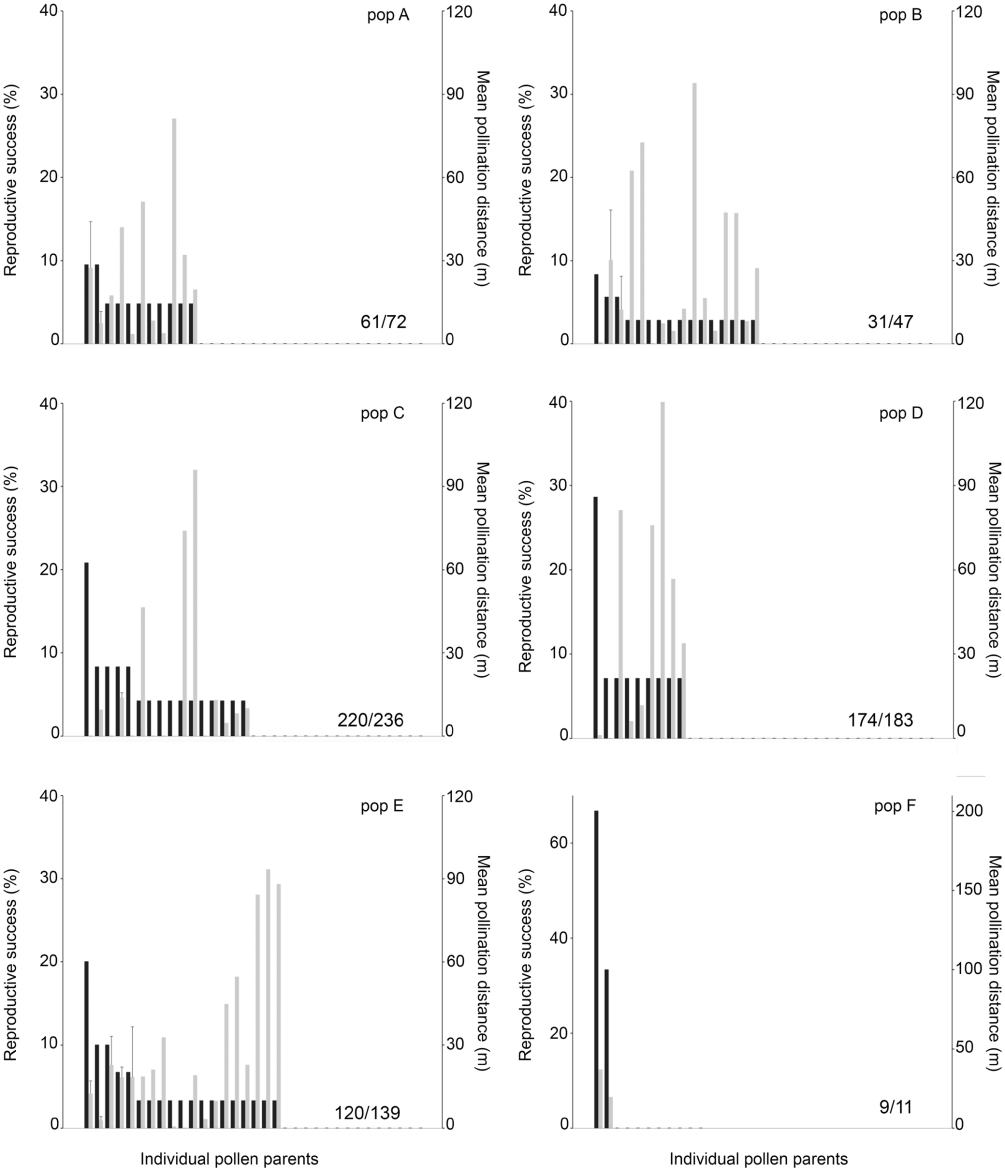
Individual male success and pollination distances in study populations of *Conospermum undulatum*. For each individual pollen parent, the percentage of inferred pollinations (black bars) and mean distances of inferred pollinations (grey bars with standard errors). In the left margin, the number of parents with null reproductive success.

Individual male reproductive success, measured as the proportion of seedlings assigned to each pollen donor, ranged between 20% and 26.8% in populations C, D and E; whereas, in the two large populations A and B, the most successful father sired 9.5% and 8.3% of the seedlings, respectively (Figure 2). Population F was excluded from this analysis due to the low number of seeds produced.

Populations were mostly similar in terms of number of pollen contributors to local offspring, showing a high number of reproductively unsuccessful males (Figure 2). The percentage of plants that were reproductively unsuccessful as pollen donors ranged between 81.8% and 95.1%, except for population B, where 31 of the 47 sampled individuals (66%) had null reproductive success.

## 4 Discussion

Gene flow is a fundamental force in determining how populations respond to anthropogenic habitat fragmentation. The recent urban expansion of Perth (Kelobonye et al. 2019) allowed us to investigate the short‐term effects of fragmentation on the pollen flow dynamics of *C. undulatum* , a species coevolved with small native bees for pollen dispersal. In this threatened species, paternity analysis showed a high frequency of short‐range pollen dispersal and revealed there is likely almost no connection among urban remnant populations, with consequent implications for conservation. This fine‐scale, comprehensive investigation complements previous studies on *C. undulatum* and provides further insight for understanding the responses of this threatened species to land‐use change.

### 4.1 Contemporary Pollen Movement

The effects of recent fragmentation are evident in the near‐complete lack of pollen immigration observed in remnant populations of *C. undulatum* . While we could not precisely quantify pollen immigration in the two large populations, A and B, the predominance of short‐distance mating, along with the presence of hundreds of ungenotyped plants surrounding the focal areas, indicates that most unassigned progeny were likely sired locally. High levels of pollen dispersal within large populations suggest that gametes could travel unimpeded through unfragmented bushland maintaining a high genetic connectivity over large distances. This capacity for pervasive pollen‐mediated gene flow likely once characterised the entire distribution range in the pre‐fragmented landscape, as shown by the weak genetic structure found in this study, and agrees with the high estimates of genetic connectivity found in a previous study of *C. undulatum* (Delnevo et al. 2021). The combination of weak genetic structure in adult plants and limited pollen immigration across remnant populations demonstrates the impact of fragmentation on the mating pattern of the species. These results support the growing body of literature demonstrating that the effects of recent habitat fragmentation in plant populations are unlikely to be detected by looking at genetic indexes in adult cohorts of plants that pre‐date recent population declines (Aguilar et al. 2019; Mimura et al. 2009; Vranckx et al. 2012). Instead, an understanding of contemporary mating patterns is needed to detect signals of reduced gene flow in fragmented remnants.

By assessing the degree of isolation among remnant populations and mapping the geographic locations of individual *C. undulatum* plants, we were able to determine local pollen dispersal patterns and minimum dispersal distances across the landscape. The analysis revealed that most pollen movement occurred within‐populations, with only population D exhibiting significant levels of pollen immigration, indicating inter‐population dispersal. It is worth noting that population D is separated from the nearest unsampled plants by ~650 m of cleared rural land, as opposed to built‐up areas (satellite image in Figure S1). This distance falls within the estimated genetic connectivity range for *C. undulatum* (~1000 m; Delnevo et al. 2021) and aligns with the inferred foraging range of its primary pollinator, *L. conospermi* (~500 m), based on the relationship between body size and foraging distance in hymenopterans (Greenleaf et al. 2007). Pollen‐mediated gene flow therefore appears to be possible between close fragments in a vegetated matrix. Whereas populations separated by built‐up residential areas, even if closer than 650 m from each other, are now completely isolated as such urban areas may generate more significant barriers to small native bees compared to honeybees and birds (Andersson et al. 2017). This reduction in contemporary inter‐population gene flow may limit the capacity of small populations to regain lost genetic diversity through connectivity with larger remnants, potentially compromising the long‐term persistence of this threatened species.

### 4.2 Selfing Rate

*Conospermum undulatum*, as in the majority of proteaceous species (Collins and Rebelo 1987; Goldingay and Carthew 1998), has been considered a self‐incompatible plant. In a recent study, Delnevo, van Etten, et al. (2019) reported that manual pollination using pollen from different flowers on the same plant resulted in an average fruit set of 26.4%, but the embryos failed to develop fully, producing no viable seeds. This indicates that self‐incompatibility in *C. undulatum* is not only a consequence of its specific floral morphology, which prevents autogamy, but also an adaptation to avoid self‐pollination between flowers on the same plant (geitonogamy). In contrast, our paternity investigation here revealed a total of 13 plants that produced selfed progeny. This may be a factor of the resprouting ability of *C. undulatum* combined with the likely longevity of plants. Analysis of 11 microsatellite loci on five branches of 54 old individuals of the resprouter *Banksia attenuata*, also Proteaceae, showed that 7.4% possessed non‐identical heterozygous alleles in different branches on the same plant (Lamont et al. 2011). Indeed, since resprouter species can survive many regeneration cycles and the accumulation of mutant alleles is simply a time‐dependent process, older individuals may be more likely to develop somatic mutations, and in some cases, this may allow self‐fertilisation between different stems of the same plant. Our results agree with this hypothesis as plants that sired selfed progeny had a much higher average number of stems compared to the average value across all populations of *C. undulatum* .

### 4.3 Conservation Implications

This study emphasises the value of integrating population genetic analyses to investigate both historical and contemporary gene flow, alongside evaluations of ecological and environmental drivers, in order to better understand the effects of recent habitat fragmentation on long‐lived plant species. Reductions in heterozygosity following declines in population size can lower fitness through inbreeding depression (Charlesworth and Willis 2009), and in predominantly outcrossing species like *C. undulatum* , such inbreeding can particularly affect early fitness traits, including embryo development and seed germination (Aguilar et al. 2019; Vranckx et al. 2012). The lack of contemporary inter‐population genetic connectivity likely due to anthropogenic barriers to pollen dispersal, as found in this study, may reduce reproductive output in isolated populations over time.

Moreover, changes in the spatial configuration of remnant populations due to land‐use alteration can disrupt pollen flow dynamics, with smaller and/or more isolated populations receiving fewer pollinator visits (Dauber et al. 2010). This general trend was observed in *C. undulatum* with reduction of pollinator visitation (Delnevo, van Etten, Byrne, et al. 2020) associated with a reduction in pollen movements decreasing the reproductive success of these mostly self‐incompatible plants. In addition, within small fragments, pollinators tend to increase the number of within‐individual floral visits (Eckert 2000), with a consequent increase in the transfer of self‐pollen between different flowers of the same individual, thus wasting reproductive effort. Despite our finding that geitonogamous selfing is possible in *C. undulatum* , it appears more likely in larger populations and cannot provide a reliable reproductive assurance in small remnant populations of this threatened species. The decline in reproductive output appeared most evident in all the three small populations selected for this study (F, G and H), where a few fruits were produced but no viable seeds developed (except in population F where we obtained three seedlings).

The relatively high heterozygosity and weak genetic structure observed in adult cohorts of *C. undulatum* suggest that selfing was not common in the pre‐fragmented landscape. It is unclear whether selfing has increased due to fragmentation or whether selfed progeny are purged at early stages of development.

Many plant species can withstand impacts of habitat fragmentation due to the presence of highly mobile pollinators that maintain substantial inter‐population pollen flow (e.g., Byrne et al. 2007, 2008). However, this is not the case for species like *C. undulatum* , which is largely self‐incompatible and depends on small native bees and ants for pollen transfer (Delnevo, van Etten, Byrne, et al. 2020; Delnevo, van Etten, Clemente, et al. 2020). Consequently, habitat changes may pose a greater threat to their long‐term persistence compared with species that have mixed mating systems and generalist pollinators. Considering species‐specific life history and ecological traits is therefore crucial for conservation planning, including translocations and reintroductions, to ensure population sizes remain sufficient to maintain genetic diversity and functional mating systems.

## Author Contributions

**Nicola Delnevo:** conceptualization (lead), formal analysis (lead), funding acquisition (lead), investigation (lead), methodology (equal), visualization (equal), writing – original draft (lead). **Andrea Piotti:** formal analysis (equal), methodology (equal), writing – original draft (equal). **Eddie J. van Etten:** investigation (equal), methodology (equal), supervision (equal), writing – review and editing (equal). **William D. Stock:** conceptualization (equal), methodology (equal), writing – review and editing (equal). **David L. Field:** writing – review and editing (equal). **Margaret Byrne:** supervision (equal), writing – review and editing (equal).

## Funding

This project was supported by The Holsworth Wildlife Research Endowment & The Ecological Society of Australia [R12019/G1004377 to ND]. This research was jointly supported by Edith Cowan University Industry Collaboration Grant and the Department of Biodiversity, Conservation and Attractions, Western Australia [G1002531].

## Conflicts of Interest

The authors declare no conflicts of interest.

## Supporting information


**Table S1:** Pairwise population matrix of *G*_ST_ values.
**Table S2:** Pairwise population matrix of *D* values.
**Figure S1:** Map showing the clear distinction between built‐up areas (north) cleared rural land (centre and south).

## Data Availability

Data are available via the Dryad Digital Repository https://doi.org/10.5061/dryad.95x69p907.
