# Supplementary material for: Exchanging Genes Within a City: Analysis of Pollen Flow Patterns in a Narrow Endemic Plant Species Threatened by Urbanisation
Source: Ecol Evol. 2026 Apr 13;16(4):e73406. doi: 10.1002/ece3.73406 (PMC13071750; doi:10.1002/ece3.73406)
Supplement: Supplementary file 1 — Table S1: Pairwise population matrix of G ST values. Table S2: Pairwise population matrix of D values. Figure S1: Map showing the clear distinction between built‐up areas (north) cleared rural land (centre and south). [file ECE3-16-e73406-s001.docx]

| Table S1. Pairwise population matrix of *G_ST_* values | | | | | | | | |
| --- | --- | --- | --- | --- | --- | --- | --- | --- |
|  | A | B | C | D | E | F | G | H |
| A | 0.000 | 0.001 | 0.001 | 0.001 | 0.001 | 0.001 | 0.001 | 0.001 |
| B | 0.013 | 0.000 | 0.001 | 0.001 | 0.001 | 0.001 | 0.001 | 0.001 |
| C | 0.023 | 0.018 | 0.000 | 0.001 | 0.001 | 0.001 | 0.001 | 0.001 |
| D | 0.012 | 0.008 | 0.019 | 0.000 | 0.001 | 0.001 | 0.001 | 0.001 |
| E | 0.007 | 0.012 | 0.022 | 0.010 | 0.000 | 0.008 | 0.001 | 0.001 |
| F | 0.018 | 0.013 | 0.021 | 0.014 | 0.009 | 0.000 | 0.001 | 0.001 |
| G | 0.051 | 0.055 | 0.076 | 0.048 | 0.049 | 0.045 | 0.000 | 0.001 |
| H | 0.083 | 0.099 | 0.111 | 0.083 | 0.081 | 0.081 | 0.083 | 0.000 |
| *G_ST_* values below the diagonal.  *P*-value based on 999 permutations above diagonal. | | | | | | | | |

| Table S2. Pairwise population matrix of *D* values | | | | | | | | |
| --- | --- | --- | --- | --- | --- | --- | --- | --- |
|  | A | B | C | D | E | F | G | H |
| A | 0.000 | 0.001 | 0.001 | 0.001 | 0.001 | 0.001 | 0.001 | 0.001 |
| B | 0.075 | 0.000 | 0.001 | 0.001 | 0.001 | 0.002 | 0.001 | 0.001 |
| C | 0.131 | 0.097 | 0.000 | 0.001 | 0.001 | 0.001 | 0.001 | 0.001 |
| D | 0.067 | 0.046 | 0.107 | 0.000 | 0.001 | 0.001 | 0.001 | 0.001 |
| E | 0.040 | 0.063 | 0.119 | 0.055 | 0.000 | 0.006 | 0.001 | 0.001 |
| F | 0.097 | 0.068 | 0.109 | 0.079 | 0.046 | 0.000 | 0.001 | 0.001 |
| G | 0.222 | 0.230 | 0.327 | 0.209 | 0.206 | 0.181 | 0.000 | 0.001 |
| H | 0.301 | 0.354 | 0.404 | 0.305 | 0.288 | 0.273 | 0.222 | 0.000 |
| *D* values below the diagonal.  *P*-value based on 999 permutations above diagonal. | | | | | | | | |


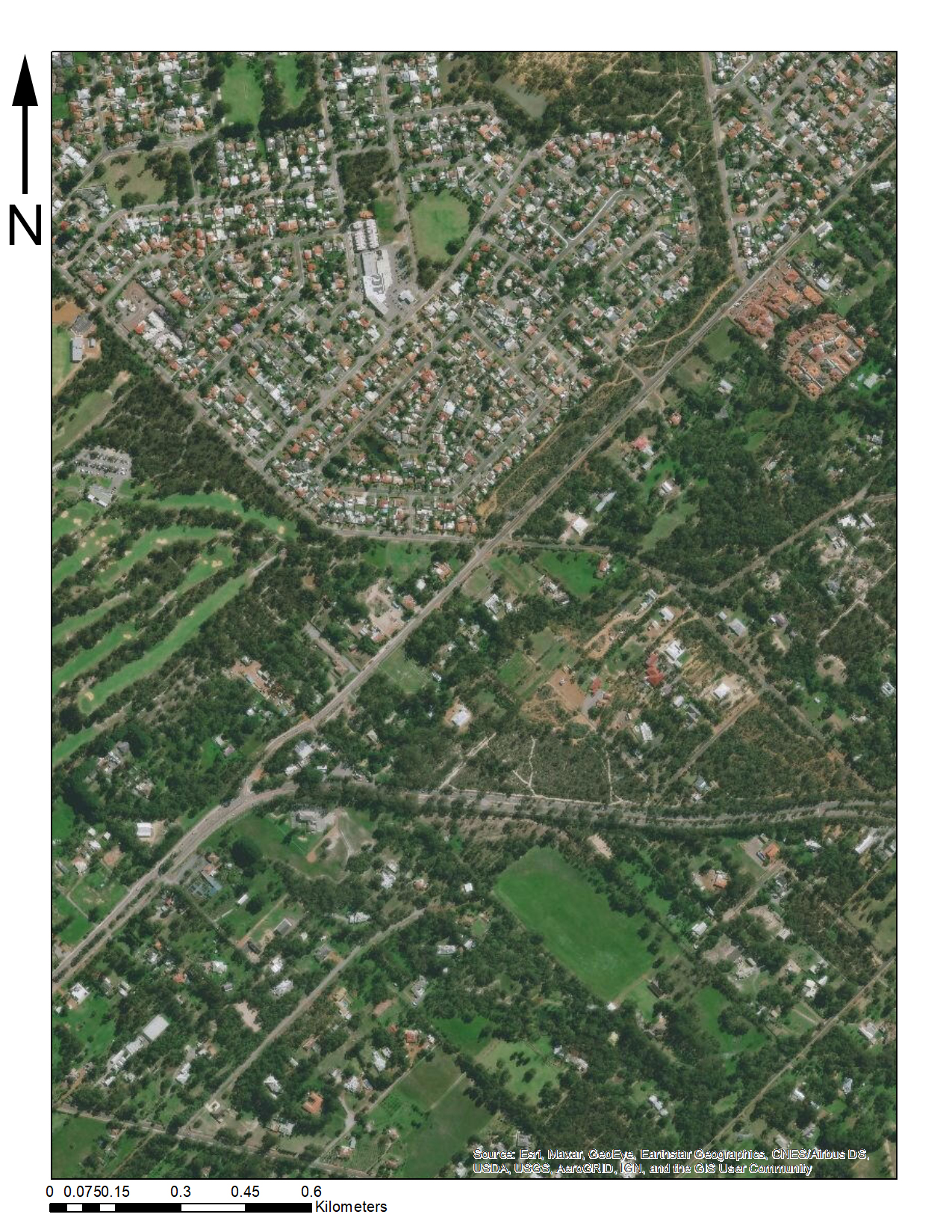


**Figure S1** Map showing the clear distinction between built-up areas (north) cleared rural land (centre and south)
